# Prenatal Exposure to Metabolism-Disrupting Chemicals, Cord Blood Transcriptome Perturbations, and Birth Weight in a Belgian Birth Cohort

**DOI:** 10.3390/ijms24087607

**Published:** 2023-04-20

**Authors:** Anran Cai, Lützen Portengen, Gökhan Ertaylan, Juliette Legler, Roel Vermeulen, Virissa Lenters, Sylvie Remy

**Affiliations:** 1 Department of Population Health Sciences, Institute for Risk Assessment Sciences, Utrecht University, 3584 CM Utrecht, The Netherlands; 2 VITO Health, Flemish Institute for Technological Research (VITO), 2400 Mol, Belgium; 3 Julius Center for Health Sciences and Primary Care, University Medical Center Utrecht, 3584 CG Utrecht, The Netherlands

**Keywords:** endocrine-disrupting chemical, transcriptomics, birth weight, epidemiology

## Abstract

Prenatal exposure to metabolism-disrupting chemicals (MDCs) has been linked to birth weight, but the molecular mechanisms remain largely unknown. In this study, we investigated gene expressions and biological pathways underlying the associations between MDCs and birth weight, using microarray transcriptomics, in a Belgian birth cohort. Whole cord blood measurements of dichlorodiphenyldichloroethylene (*p,p’*-DDE), polychlorinated biphenyls 153 (PCB-153), perfluorooctanoic acid (PFOA), perfluorooctane sulfonic acid (PFOS), and transcriptome profiling were conducted in 192 mother–child pairs. A workflow including a transcriptome-wide association study, pathway enrichment analysis with a meet-in-the-middle approach, and mediation analysis was performed to characterize the biological pathways and intermediate gene expressions of the MDC–birth weight relationship. Among 26,170 transcriptomic features, we successfully annotated five overlapping metabolism-related gene expressions associated with both an MDC and birth weight, comprising *BCAT2*, *IVD*, *SLC25a16*, *HAS3*, and *MBOAT2*. We found 11 overlapping pathways, and they are mostly related to genetic information processing. We found no evidence of any significant mediating effect. In conclusion, this exploratory study provides insights into transcriptome perturbations that may be involved in MDC-induced altered birth weight.

## 1. Introduction

Metabolism-disrupting chemicals (MDCs) have been defined as natural or anthropogenic endocrine-disrupting chemicals (EDCs) that can promote metabolic changes and ultimately lead to obesity, type 2 diabetes and/or non-alcoholic fatty liver disease (NAFLD) [1]. In line with the Developmental Origins of Health and Disease (DOHaD) hypothesis [1], the prenatal period is a highly sensitive and vulnerable phase during which stressors, such as MDCs, can alter cell numbers and fate, gene expression, and protein levels that may lead to changes in tissue and organ function and contribute to increased susceptibility to a variety of non-communicable diseases later in life [2]. This may be the result of differences in toxicokinetics between children and adults and from time-dependent programming during early development [3].

Both high and low birth weight (HBW and LBW) are considered important predictors of later perturbed metabolic outcomes in children and adults [4,5,6]. While some observational studies have demonstrated associations between exposure to MDCs [including dichlorodiphenyldichloroethylene (*p,p’*-DDE), polychlorinated biphenyl-153 (PCB-153), perfluorooctanoic acid (PFOA), and perfluorooctane sulfonic acid (PFOS)] and birth weight [7,8,9,10], the molecular mechanisms of action remain poorly understood. The field of omics, based on high-throughput biochemical data, provides promising opportunities to advance and enhance our understanding of the impact of MDCs on child health, including by revealing changes in the gene expression using transcriptome profiling [11,12].

Assessing the effects of various chemical exposures on gene expression may help to uncover cellular mechanisms through which exposures influence the development of metabolic disorders in human populations. Several recent epidemiological studies using transcriptomics data have increased our understanding of how exposure to MDCs may perturb gene expression, and have identified regulatory pathways that may be affected by these exposures [13,14,15], as well as links between gene expression and birth weight [16,17,18,19]. However, to our knowledge, a study assessing the transcriptome in relation to both MDCs and birth weight in the same study population has not been performed.

Based on results from our previous birth cohort study [15], several MDCs (*p,p’*-DDE, PCB-153, PFOA, and PFOS) were suggested to play a role in transcriptional changes which are related to metabolic health outcomes. This led us to hypothesize that prenatal exposure to MDCs induces transcriptional modifications that, in turn, affect birth weight and have adverse effects on human health. Here, we aim to identify transcriptomic alterations in the cord blood of Belgian mother–child pairs that are associated with both prenatal MDC levels and birth weight in order to better understand the molecular effects and the underlying mechanisms.

## 2. Results

### 2.1. Population Characteristics

Demographic and exposure information for participants are shown in Table 1 and Table S1. The median gestational age was 40 weeks. Most children (98%) had a birth weight at or more than 2500 g, with a median of 3540 g. The median concentrations were 75.9 ng/g lipid, 28.7 ng/g lipid, 1600 ng/L, and 2700 ng/L for *p,p’*-DDE, PCB-153, PFOA, and PFOS, respectively (Table S1). The majority of the mothers had completed a high level of education (59%), had a normal pre-pregnancy body mass index (BMI) between 18.5 and 25 kg/m^2^ (71%), and did not smoke during pregnancy (85%). In addition, 38% of mothers were nulliparous and 57% were above 30 years of age at delivery.

### 2.2. Gene Expression Associated with MDCs and Birth Weight

Using a transcriptome-wide association study (TWAS) approach, we failed to select any features from models (1) or (2) with significance levels of false discovery rate (FDR) <0.05 or 0.20, and selected only a few features with a stringent *p*-value < 0.01 (Table 2). In order to avoid excluding weak but possibly relevant features, we used a relatively lenient *p*-value < 0.05 to select features for further analyses as an exploratory study. With *p*-value < 0.05, we found that 2110 out of 26,170 features were associated with one or more MDCs (777, 623, 333, and 624 for *p,p’*-DDE, PCB-153, PFOA, and PFOS, respectively; Table 2), and 775 features were associated with birth weight. A similar number of associated features were found in the sensitivity analyses of gestational age-unadjusted MDC–transcriptome associations (Table S2). In addition, as shown in the volcano plots (Figure S1a–e), the significance and directionality of gene expression obtained with and without adjustment for gestational age were consistent in the TWAS models for MDCs and features.

**Figure 1:**
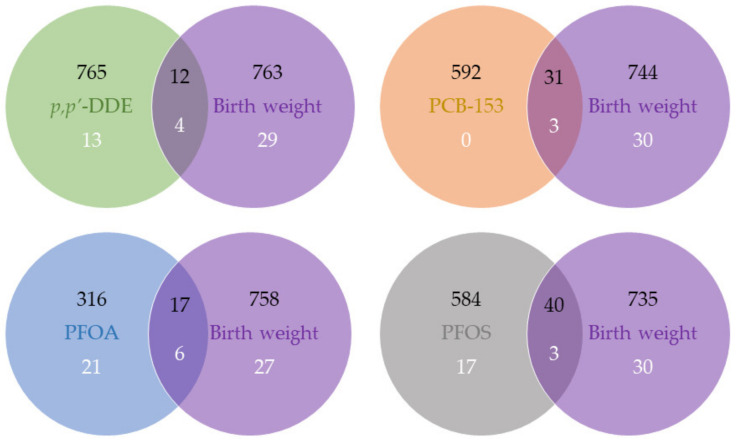
Venn diagram of features and enriched pathways associated with MDCs and birth weight. Number in black refers to the number of features at *p*-value < 0.05, and number in white refers to the number of enriched pathways at FDR < 0.05.

At *p*-value < 0.05, we found overlapping features associated with an MDC (*p,p’*-DDE, PCB-153, PFOA, or PFOS) and birth weight (12, 31, 17, and 40, respectively; Figure 1). These features were annotated to corresponding unique gene symbols, and according to the Human Protein Atlas and GeneCards [20,21], several were components of metabolism-related pathways, including *branched-chain aminotransferase 2* (*BCAT2)* (amino acid metabolism; valine, leucine, and isoleucine degradation; valine, leucine, and isoleucine biosynthesis; and pantothenate and CoA biosynthesis), *isovaleryl-CoA dehydrogenase (IVD)* (valine, leucine, and isoleucine degradation), *solute carrier family 25-A16 (SLC25A16)* (pantothenate and CoA biosynthesis), *Hyaluronan Synthase 3 (HAS3)* (carbohydrate metabolism and glycosaminoglycan metabolism), and *Membrane Bound O-Acyltransferase Domain Containing 2 (MBOAT2)* (glycerophospholipid metabolism) (Table 3). However, with the mediation analysis, we did not observe any overlapping gene expression playing a significant mediating role, given the relatively large FDR values (Table 3). In addition, the individual associations of these five gene expressions with an MDC or birth weight is shown in Table S3.

### 2.3. Pathways Associated with MDCs and Birth Weight

The MDC- or birth weight-associated pathways at FDR < 0.05 with at least five genes involved are represented in Table S4. There were 17, 3, 27, 20, and 33 pathways associated with *p,p’*-DDE, PCB-153, PFOA, PFOS, and birth weight, respectively; most of them were related to genetic information processing and organismal systems. Notably, one metabolic pathway [glycosaminoglycan biosynthesis] was linked to *p,p’*-DDE; three [metabolism of xenobiotics by cytochrome P450, drug metabolism, and type 1 diabetes mellitus (T1D)] were linked to PFOA, two [amino sugar and nucleotide sugar metabolism, type I diabetes mellitus] were linked to PFOS, and five [oxidative phosphorylation (OXPHOS), non-alcoholic fatty liver disease (NAFLD), cysteine and methionine metabolism, sulfur metabolism, and valine, leucine, and isoleucine degradation] were linked to birth weight.

At FDR < 0.05, we found that four, three, six, and three pathways associated with birth weight ovrlapped with *p,p’*-DDE, PCB-153, PFOA, and PFOS, respectively (Figure 1). They mostly belong to the “genetic information processing” category in the Kyoto Encyclopedia of Genes and Genomes (KEGG) Pathway Database [22], and none of them were metabolism-related pathways (Table 4). The PC1 scores used to represent pathways in the mediation analysis explained 37–57% of the variance in the involved genes, and given the insignificant average causal mediation effects (ACMEs) with large FDR values, we did not observe any pathway that mediated both MDC and birth weight (Table 4).

## 3. Discussion

Transcriptome changes in early life may act in response to environmental exposures and subsequently lead to adverse health outcomes later in life; however, epidemiological studies are scarce. This is the first paper that evaluated the cord blood transcriptome with MDC exposures and birth weight. We examined differences in transcriptomics at the gene and pathway levels.

The five gene expressions that are metabolism-related and were found to be associated with both an MDC and birth weight are *BCAT2, IVD, SLC25a16, HAS3,* and *MBOAT2*. Birth weight may be altered by an MDC through one of these gene expressions, although we did not find a mediating effect to be significant. Branched-chain amino acids (BCAAs) are associated with the progression of obesity-related metabolic disorders [23]; additionally, BCAA catabolism is suggested to play a role in the pathogenesis of metabolic disturbances, and BCAT2 is an important enzyme that catalyzes the initial step of BCAA catabolism [24,25]. In a recent human study [26], *BCAT2* variants were detected in Spanish infants suspected of having maple syrup urine disease—a rare metabolic disorder that some babies are born with. In line with our finding on higher *BCAT2* expression with high birth weight, LBW pigs were found to express less *BCAT2* mRNA in the longissimus dorsi muscle compared to normal birth weight pigs [27]. Similarly, higher *BCAT2* mRNA was revealed in the blastocysts of diabetic rabbits compared to control blastocysts [28]. We observed an inverse association of *IVD* expression with birth weight. It has been demonstrated that the deficiency of the mitochondrial enzyme *IVD* may lead to isovaleric acidemia (IVA), an inherited metabolic disorder that may cause problems with the breakdown of the amino acid leucine [29]. Children with this condition may fail to gain weight and often experience developmental delays [30]. *SLC25a16* has been considered as a carrier of Grave’s disease, which causes hypothyroidism [31]. On the other hand, hypothyroidism is thought to cause HBW [32,33], which may explain the association we found between *SLC25a16* and higher birth weight, but this needs to be further explored. Our results on gene expression suggest new insights into birth weight changes indirectly caused by MDCs, and also provide some support, albeit weak signals, for the existing evidence from transcriptomics–birth weight research. However, it is also important to note that none of the features selected for further analyses from the TWAS models passed the FDR correction threshold; our gene expression results should therefore be viewed as exploratory and hypothesis-generating.

Metabolism-related pathways linked to both an MDC and birth weight were not observed in this study. However, some results on the metabolism-related pathways associated with an MDC or birth weight are noteworthy. For PFOA, we have observed positive associations with the metabolism of xenobiotics by cytochrome P450 and drug metabolism and inverse association with T1D. In a mouse study, PFOA was found to induce the cytochrome P450 enzyme by activating constitutive androstane receptor (CAR) nuclear receptors [34]. Another mouse study has shown that PFOA may induce drug metabolism, and then lead to liver injury [35]. For PFOS, we observed inverse associations with amino sugar and nucleotide sugar metabolism, and T1D. Consistently, PFOS-induced altered amino sugar and nucleotide sugar metabolism were found in a recent zebrafish study, as well as in Hispanic children [36,37]. In a large U.S. study, PFOA and PFOS were associated with a reduced risk of T1D in adults [38], but in a recent Finnish study, they both were associated with an increased risk of T1D in newborns [39]. For birth weight, lower birth weight was found to be associated with six metabolism-related pathways, comprising OXPHOS, NAFLD, cysteine and methionine metabolism, sulfur metabolism, valine, leucine and isoleucine degradation, and fatty acid biosynthesis. Consistent with our findings, LBW was shown to be associated with OXPHOS in the skeletal muscle and myotubes of Danish individuals [40,41]. A study investigating the relationship between birth weight and NAFLD, in 538 children, also showed an overrepresentation of LBW in those with NAFLD compared with the general U.S. population [42]. This inverse relationship between birth weight and NAFLD occurrence was also confirmed in a large French prospective cohort study of 55,034 adults [43]. Likewise, a recent systematic review and meta-analysis demonstrated that excess methionine and cysteine led to lower birth weight [44]. The effect of branched chain amino acids (valine, leucine and isoleucine) on birth weight was not yet clear, and most of the existing studies were animal studies [44]. In addition, there is growing evidence that there may be an association between high fatty acid levels and LBW [45,46,47,48].

The strengths of our study include the well-defined sampling frame and the use of omics techniques, which allow for the investigation of multiple genes and pathways simultaneously, in order to explore the impact of MDCs on the transcriptome perturbations and the subsequent impact on the birth weight. We also acknowledge several limitations of this study. First, the relatively small sample size (*n* = 193 mother–child pairs) of our study population was prone to modest statistical power in detecting associations. Also for this reason, we did not perform sex-specific analysis despite that EDCs have been shown to exert different adverse effects in males and females, both in laboratory animals and in humans [49]. Second, it should also be noted that the concentrations of *p,p’*-DDE, PCB-153, and PFOS in our study population were relatively low compared with the median exposure levels observed in other studies that found associations with birth weight [7,50,51], and they may not have been high enough to have a measurable effect, or the limited contrast in exposures may have limited statistical power to detect associations; PFOA levels were more comparable with levels in other studies. Third, the cross-sectional design of the study precluded establishing a temporal or causal relationship between MDC concentrations, transcriptome, and birth weight. Last, as with any other observational epidemiological study, there may be residual confounding bias due to uncontrolled unmeasured confounders, but we expected these to be minimal, as we carefully adjusted for a set of covariates that have been shown to be important with the help of directed acyclic graphs (DAGs).

In addition, the mechanisms are complex and sensitive windows, for exposure to MDCs may vary depending on the specific chemical. Alterations at the molecular level caused by MDCs may also differ according to the specific outcome being studied. Therefore, different exposure windows and outcomes should be assessed in further studies investigating the metabolism-disrupting effects of chemicals.

## 4. Materials and Methods

### 4.1. Study Population

We used data from the second cycle of the Flemish Environment and Health Study (FLEHS II, 2008–2009), whose design and recruitment have been previously described in detail [52]. In short, 255 mother–child pairs were recruited from Flanders, Belgium, using a two-stage sampling procedure, with provinces as the primary sampling unit and maternity units as the secondary sampling unit. Mothers who had lived for at least 10 years in Flanders and were able to fill in Dutch questionnaires were invited to participate. The number of participants in each province was proportional to the number of inhabitants. Among the mother–child pairs, 195 were randomly selected for transcriptome profiling. We restricted our analyses to the 193 term births (gestational age ≥37 weeks) in this study because preterm birth is a potential mediator of the effects of chemical exposures on birth weight [53].

### 4.2. Exposure Assessment

Several classes of environmental chemicals were measured in cord blood samples. Here, we have focused our analyses on MDC exposures that could be detected in at least 60% of the cord blood samples [54]: *p,p’*-DDE, PCB-153, PFOA, and PFOS (see Supplementary Material, Table S1 for detection rates, which ranged from 97 to 100% for these four chemicals). Samples were collected immediately after birth and stored at −80 °C until the measurements. MDC concentrations were measured using gas chromatography-electron capture negative ionization mass spectrometry (for *p,p’*-DDE and PCB-153) and high-performance liquid chromatography with tandem mass spectrometry detection (for PFOA and PFOS), as previously described [55,56]. All of the samples had quantifiable concentrations of *p,p’*-DDE, PFOA, and PFOS, while for PCB-153, 3% of the samples had values below the limit of quantification (LOQ, 300 ng/L). These values were then imputed using maximum likelihood estimation, assuming a censored log-normal distribution for values above the LOQ and conditional on the observed values for other biomarkers [54,57]. Lipid-standardized *p,p’*-DDE and PCB-153 concentrations were calculated based on estimated total lipids [*total lipids = 50.49 + 1.32 × (cholesterol + triglycerides) (mg/dL)*] and expressed as ng/g lipids for subsequent analyses [15]. All MDC concentrations were log_2_-transformed in order to reduce the potential influence of extreme values.

### 4.3. Transcriptome Profiling and Processing

As previously described [15,58], total RNA was extracted from the cord blood samples and stored at −80 °C. Amplified and labeled cRNA were then hybridized to 4 × 44 K Agilent Whole Human Genome Microarray (design 014850, one-color experimental setup with Cy3-labeling; Santa Clara, CA, USA), according to the manufacturer’s protocol. Preprocessing, quality assessment, and normalization of the microarray data were performed as described previously [15]. Briefly, the arrays were scanned with an Agilent scanner (G2565BA) and were subjected to primary quality control using the Agilent Feature Extraction Software (Version 10.7; Santa Clara, CA, USA). Furthermore, for each feature on the array, the quantile-normalized and log2-transformed signal intensity derived from Cy3 fluorescent dye was used for subsequent analyses. For replicated features on the array, the mean of signal intensities was calculated. After control and noise filtering by removing features with signal intensity below 3, 33,543 features retained. Thereafter, we used the R package *Combat* to eliminate possible batch effects related to different hybridization dates (28 dates from 14 September 2011 to 11 January 2012) [59,60]. Lastly, 26,170 (78.02%) features were annotated to a total of 17,880 unique gene symbols according to the Molecular Signatures Database (MSigDB) and were subjected to further statistical and functional analyses [61].

### 4.4. Outcome Assessment and Covariates

We considered birth weight (g) as our outcome of interest. DAGs were used to guide the selection of covariates (Figures S1b and S2a). The set of minimally sufficient covariates included sex of the child (girl, boy), smoking during pregnancy (smoking, non-smoking), parity (0, 1, ≥2), maternal education (low, medium, high), maternal age at delivery (<27, 27 < 30, 30 < 33, ≥33 years), pre-pregnancy BMI (<18.5, 18.5 < 25, 25 < 30, ≥30 kg/m^2^), and gestational age (weeks). Birth weight and child sex were collected from maternity medical records. Other covariate data was obtained from questionnaires. Missing data in covariates and exposures that were completely missing (1–3% and 1% of participants had one of more missing values, respectively) were singly imputed using the R package *mice* [62].

### 4.5. TWAS

TWASs were conducted in order to investigate the association of global transcriptomics with (1) MDCs and (2) birth weight. We used the following multivariable linear models to evaluate the effects of MDC exposures and potential predictors of birth weight, for each feature and MDC separately:

*log_2_(feature intensity_i_) = β_0_ + β_1_ log_2_(MDC_i_) + β_2_ sex_i_ + β_3_ smoking during pregnancy_i_ + β_4_ parity_i_ + β_5_ education_i_ + β_6_ age at delivery_i_ + β_7_ pre-pregnancy BMI_i_ + β_8_ gestational age_i_ + ε_1i_* (1)

*birth weight_i_ = γ_0_ + γ_1_ log_2_(feature intensity_i_) + γ_2_ sex_i_ + γ_3_ smoking during pregnancy_i_ + γ_4_ parity_i_ + γ_5_ education_i_ + γ_6_ age at delivery_i_ + γ_7_ pre-pregnancy BMI_i_ + γ_8_ gestational age_i_ + ε_2i_* (2)

where *i* indexes the study subjects and Model (1) describes the association between a single transcriptomic feature and a single MDC, while Model (2) describes the association between birth weight and a single transcriptomic feature. Parameters *β_0_* and *γ_0_* are the model intercepts, while *β_1_* and *γ_1_* refer to the effect estimates (slopes) for a single MDC on a single transcriptomic feature, and for a single transcriptomic feature on birth weight, respectively. Parameters *β_2–8_* and *γ_2–8_* are coefficients corresponding to other covariates in the model, and *ε_1i_* and *ε_2i_* represent the residual errors, which are assumed to follow a normal distribution.

According to observed *p*-values for *β_1_* and *γ_1_*, we estimated FDR using the method of Benjamini and Hochberg to correct for multiple testing and to select significant features [63].

### 4.6. Enrichment Pathway Analysis

In order to find pathways associated with MDC exposures and birth weight, we carried out Gene Set Enrichment Analyses (GSEA) using the WEB-based GEne SeT AnaLysis Toolkit (WebGestalt; Los Angeles, CA, USA) tool with pathway gene sets from the KEGG database [22,64,65]. First, we generated the respective ranked lists of all 26,170 features, sorted by their degree of differential expression (log_2_-fold change) in cord blood in relation to MDCs and birth weight, i.e., *β_1_* and *γ_1_* obtained from Models (1) and (2) [66,67]. Subsequently, the normalized enrichment scores were calculated, reflecting the degree to which pathways were enriched by ranked genes, where positive and negative values represent positive and inverse associations of pathways with MDCs or birth weight, respectively [68]. We restricted to pathways with at least five genes involved, and estimated the statistical significance using 1000 gene set permutations with FDR correction for multiple testing. Pathways with FDR < 0.05 were considered significant.

### 4.7. Mediation Analysis

**Figure 2:**
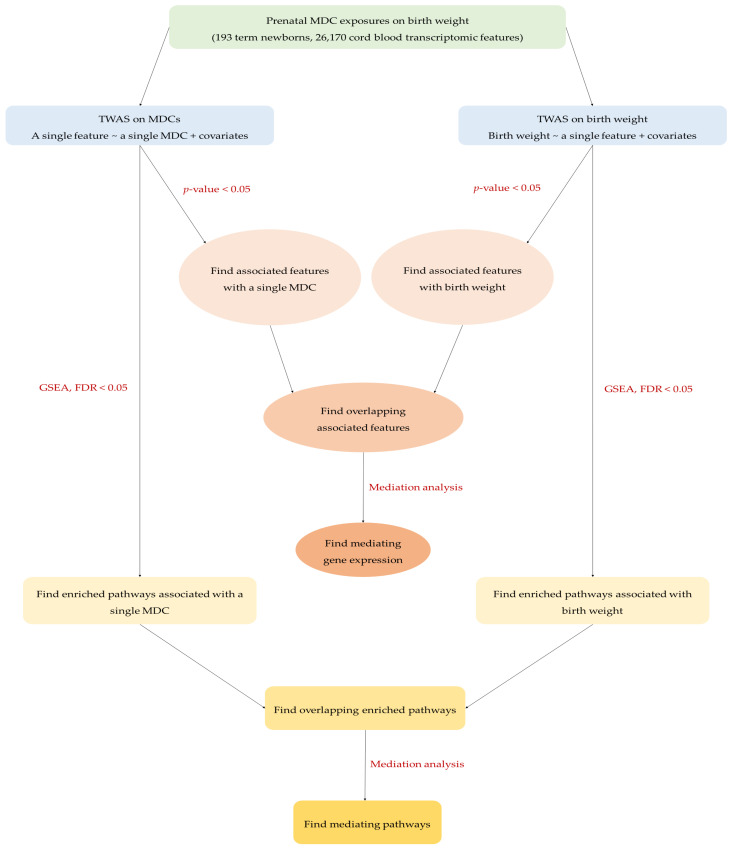
The workflow of meet-in-the-middle approach in the present study.

Figure 2 outlines the workflow of the meet-in-the-middle approach used in this study [69]. The overlapping selected features and pathways observed in association with any of the four MDCs and birth weight were further explored by mediation analysis using the R package *mediation* [70] to explore potential biological mechanisms and mediating effects linking exposure and outcome. When assessing an overlapping feature as a mediator, we included it in the mediation model, and computed ACMEs (also known as indirect effects) using 1000 bootstrapped samples with FDR correction, and it was considered as a potential mediating feature if the FDR < 0.05. When examining an overlapping pathway as a mediator, we first performed a principal component analysis (PCA) on the genes belonging to that pathway, and then used the first principal component score (PC1) to represent that pathway in the mediation model [71,72]. ACMEs with FDR < 0.05 were generated to identify potential mediating pathways.

### 4.8. Sensitivity Analysis

Recognizing that gestational age could be associated with MDC exposure and impact transcriptome levels [15], combined with several other studies also showing that the transcriptome was substantially influenced by gestational age [73,74], gestational age was included as a control variable in our primary regression model of MDCs and transcriptome (TWAS Model (1)). On the other hand, the causal direction of the association between gestational age and MDC is not entirely clear, and it is possible that gestational age mediates the outcome [75]. Therefore, in a sensitivity analysis, we assessed MDC and transcriptome associations without adjusting for gestational age in order to avoid adjustment for a potential mediator [53].

All statistical analyses were performed in R version 4.1.0 [76].

## 5. Conclusions

In summary, we integrated cord blood TWASs in order to identify gene expressions and pathways associated with MDCs and birth weight. Taken together, our study suggested five gene expressions associated with at least one MDC and birth weight. This provides insight into the etiology of higher and lower birth weight and possible later metabolic disorders, but again, this is an exploratory study with weak signals. In order to validate our results and further understand the potential link between MDC exposures and birth weight, and to elucidate the underlying mechanisms, studies with larger sample sizes and prospective study designs combined with advanced omics techniques are warranted.

## Figures and Tables

**Table 1:** Study population characteristics of 193 mother–child pairs, Flanders, Belgium.

Characteristics	
**[*n* (%) or Median (P25–P75)]**	
**Mother**	
**Education**	
**Low**	19 (10)
**Median**	58 (30)
**High**	114 (59)
**Missing**	2 (1)
**Parity**	
**0**	74 (38)
**1**	64 (33)
**≥2**	54 (28)
**Missing**	1 (1)
**Smoking during pregnancy**	
**Non-smoking**	164 (85)
**Smoking**	24 (12)
**Missing**	5 (3)
**Age at delivery (years)**	
**<27**	35 (18)
**27 < 30**	49 (25)
**30 < 33**	57 (30)
**≥33**	52 (27)
**Pre-pregnancy BMI (kg/m^2^)**	
**<18.5**	12 (6)
**18.5 < 25**	137 (71)
**25 < 30**	28 (15)
**30**	14 (7)
**Missing**	2 (1)
**Child**	
**Sex, *n* (%)**	
**Boy**	96 (50)
**Girl**	97 (50)
**Gestational age (weeks)**	40.0 (39.0–40.0)
**Missing**	3 (2)
**Birth weight (g)**	3540 (3200–3775)
**<2500**	3 (2)
**≥2500**	190 (98)

Abbreviations: BMI, body mass index; P, percentile.

**Table 2:** Number of features associated with MDCs and birth weight at different significance levels.

	FDR < 0.05	FDR < 0.20	*p*-Value < 0.01	*p*-Value < 0.05
*p,p’*-DDE	0	0	138	777
PCB-153	0	0	75	623
PFOA	0	0	23	333
PFOS	0	0	79	624
Birth weight	0	0	162	775

Abbreviations: MDCs, metabolism-disrupting chemicals; *p,p’*-DDE, dichlorodiphenyldichloroethylene; PCB-153, polychlorinated biphenyl 153; PFOA, perfluorooctanoic acid; PFOS, perfluorooctane sulfonic acid; FDR, false discovery rate.

**Table 3:** The ACMEs of an MDC on birth weight via overlapping gene expression.

		*p,p’*-DDE		
ProbeID	GeneSymbol	GeneTitle	ACME (95% CI, g)	FDR
A_32_P223173	*MYO5BP2*	myosin VB pseudogene 2	15.56 (1.89, 34.40)	0.08
A_23_P154522	*MTA3*	metastasis associated 1 family member 3	12.81 (−5.75, 41.79)	0.22
A_24_P303524	*MICALL2*	MICAL like 2	11.37 (−0.16, 28.40)	0.08
A_23_P46369	*RAB13*	RAB13, member RAS oncogene family	11.17 (−0.10, 28.29)	0.08
A_23_P435002	*SRFBP1*	serum response factor binding protein 1	10.95 (−0.66, 28.67)	0.08
A_23_P90163	*BCAT2*	branched chain amino acid transaminase 2	−9.89 (−28.87, 1.29)	0.14
A_23_P356694	*DEFB123*	defensin beta 123	−13.10 (−34.55, 0.80)	0.10
A_32_P226186	*KIAA1549*	KIAA1549	−13.30 (−34.77, −0.28)	0.08
A_32_P126375	*NHS*	NHS actin remodeling regulator	−13.52 (−33.89, 0.20)	0.08
A_23_P101240	*VSIG10L*	V-set and immunoglobulin domain containing 10 like	−13.91 (−34.95, −0.17)	0.08
A_23_P70566	*FKBPL*	FKBP prolyl isomerase like	−15.87 (−36.64, −2.04)	0.08
A_24_P33014	*DACT3*	disheveled binding antagonist of beta catenin 3	−18.48 (−49.89, 0.22)	0.08
		**PCB-153**		
**ProbeID**	**GeneSymbol**	**GeneTitle**	**ACME (95% CI, g)**	**FDR**
A_23_P213458	*BTF3*	basic transcription factor 3	19.21 (1.21, 45.76)	0.18
A_23_P129322	*IVD*	isovaleryl-CoA dehydrogenase	15.87 (−3.19, 47.22)	0.18
A_24_P816777	*UBL7-DT*	UBL7 divergent transcript	14.79 (−1.48, 39.40)	0.18
A_24_P941051	*CSTF2T*	cleavage stimulation factor subunit 2 tau variant	14.20 (−1.75, 39.92)	0.18
A_24_P383080	*SRRT*	serrate, RNA effector molecule	14.07 (−1.18, 37.84)	0.18
A_23_P1043	*INAVA*	innate immunity activator	14.06 (−2.30, 41.12)	0.18
A_24_P2093	*XAB2*	XPA binding protein 2	13.86 (−4.94, 41.67)	0.21
A_23_P170352	*MRPL12*	mitochondrial ribosomal protein L12	13.57 (−2.58, 36.47)	0.18
A_23_P101972	*CAPN13*	calpain 13	12.90 (−0.90, 33.91)	0.18
A_23_P208167	*FPR3*	formyl peptide receptor 3	−14.25 (−40.03, 2.80)	0.18
A_23_P66311	*DNASE1*	deoxyribonuclease 1	−14.86 (−68.16, 20.26)	0.47
A_32_P174365	*SATB2*	SATB homeobox 2	−15.07 (−46.51, 3.80)	0.20
A_24_P42001	*IGSF3P2*	pseudogene similar to part of immunoglobulin superfamily 3	−15.29 (−45.26, 2.47)	0.18
A_23_P45864	*TNR*	tenascin R	−15.51 (−52.01, 7.40)	0.27
A_23_P156697	*ABHD16A*	abhydrolase domain containing 16A, phospholipase	−15.71 (−55.54, 10.26)	0.30
A_32_P109777	*PHBP9*	prohibitin pseudogene 9	−15.74 (−70.68, 20.32)	0.43
A_23_P218584	*BCL11A*	BAF chromatin remodeling complex subunit BCL11A	−16.11 (−47.21, 2.36)	0.18
A_24_P934800	*ERI2*	ERI1 exoribonuclease family member 2	−17.04 (−65.34, 13.98)	0.35
A_24_P609323	*ZNF213-AS1*	ZNF213 antisense RNA 1 (head to head)	−17.27 (−61.75, 9.59)	0.30
A_23_P125147	*RAB28*	RAB28, member RAS oncogene family	−17.49 (−44.99, 1.40)	0.18
A_23_P68922	*MICALL1*	MICAL like 1	−18.85 (−58.74, 4.03)	0.21
A_23_P210400	*KCNQ2*	potassium voltage-gated channel subfamily Q 2	−20.10 (−49.17, −0.69)	0.18
A_24_P186497	*GTF2IRD2*	GTF2I repeat domain containing 2	−20.28 (−65.43, 7.02)	0.22
A_23_P323196	*MDS2*	myelodysplastic syndrome 2 translocation associated	−20.80 (−59.81, 4.88)	0.18
A_23_P343808	*SOS1*	SOS Ras/Rac guanine nucleotide exchange factor 1	−21.46 (−60.91, 1.96)	0.18
A_32_P74075	*SLC25A16*	solute carrier family 25 member 16	−23.23 (−59.66, 0.62)	0.18
A_23_P16275	*TSKS*	testis specific serine kinase substrate	−23.31 (−61.08, 1.28)	0.18
A_23_P88466	*NPAP1*	nuclear pore associated protein 1	−24.11 (−65.66, 1.64)	0.18
A_24_P33014	*DACT3*	disheveled binding antagonist of beta catenin 3	−25.38 (−75.74, 2.97)	0.18
A_32_P149640	*EPHA5*	EPH receptor A5	−25.63 (−59.68, −1.67)	0.18
A_23_P49539	*BAHCC1*	BAH domain and coiled-coil containing 1	−27.18 (−73.81, 2.40)	0.18
		**PFOA**		
**ProbeID**	**GeneSymbol**	**GeneTitle**	**ACME (95% CI, g)**	**FDR**
A_23_P426511	*ZGRF1*	zinc finger GRF-type containing 1	27.81 (−11.07, 80.77)	0.17
A_24_P173754	*C1orf21*	chromosome 1 open reading frame 21	25.95 (−2.29, 65.53)	0.12
A_23_P149668	*KIF14*	kinesin family member 14	25.64 (1.21, 59.18)	0.11
A_23_P35977	*PDZD3*	PDZ domain containing 3	25.23 (0.14, 65.29)	0.11
A_23_P19723	*BMP5*	bone morphogenetic protein 5	24.29 (−6.57, 70.05)	0.14
A_24_P383080	*SRRT*	serrate, RNA effector molecule	22.77 (1.52, 51.40)	0.11
A_23_P133956	*KIFC1*	kinesin family member C1	22.25 (1.91, 52.27)	0.11
A_23_P128956	*ZFYVE1*	zinc finger FYVE-type containing 1	21.97 (0.10, 53.12)	0.11
A_23_P258377	*ERC1*	ELKS/RAB6-interacting/CAST family member 1	20.90 (−1.37, 53.51)	0.11
A_32_P148199	*VPS54*	VPS54 subunit of GARP complex	19.84 (−1.28, 52.35)	0.11
A_23_P329962	*SUN3*	Sad1 and UNC84 domain containing 3	19.80 (−12.34, 67.49)	0.21
A_23_P357229	*HAS3*	hyaluronan synthase 3	19.47 (0.69, 48.19)	0.11
A_23_P332413	*SLFN13*	schlafen family member 13	18.66 (−3.99, 50.40)	0.13
A_23_P94840	*DYNLRB2*	dynein light chain roadblock-type 2	−19.33 (−53.39, 0.29)	0.11
A_23_P147255	*PCBP3*	poly(rC) binding protein 3	−22.98 (−56.91, 0.65)	0.11
A_32_P208076	*ITGA2*	integrin subunit alpha 2	−25.58 (−61.58, −2.19)	0.11
A_23_P89030	*C16orf95*	chromosome 16 open reading frame 95	−28.31 (−65.10, −3.43)	0.11
		**PFOS**		
**ProbeID**	**GeneSymbol**	**GeneTitle**	**ACME (95% CI, g)**	**FDR**
A_23_P4007	*FXR2*	FMR1 autosomal homolog 2	22.03 (2.59, 48.02)	0.17
A_24_P919279	*ZNF790*	zinc finger protein 790	21.33 (−0.11, 57.21)	0.17
A_23_P143514	*SSR4P1*	signal sequence receptor subunit 4 pseudogene 1	21.11 (−9.85, 65.02)	0.23
A_23_P214727	*GPR63*	G protein-coupled receptor 63	19.46 (−1.99, 55.15)	0.17
A_24_P325046	*ZCCHC7*	zinc finger CCHC-type containing 7	19.25 (−9.60, 62.98)	0.23
A_23_P158349	*RABL3*	RAB, member of RAS oncogene family like 3	19.08 (−0.78, 47.62)	0.17
A_32_P148199	*VPS54*	VPS54 subunit of GARP complex	18.42 (1.01, 40.65)	0.17
A_23_P426511	*ZGRF1*	zinc finger GRF-type containing 1	18.30 (−14.27, 71.54)	0.30
A_24_P922808	*DESI2*	desumoylating isopeptidase 2	18.16 (−5.59, 56.98)	0.18
A_23_P78302	*NFE2L1*	nuclear factor, erythroid 2 like 1	17.83 (−11.92, 63.48)	0.29
A_24_P98086	*GNA12*	G protein subunit alpha 12	17.07 (3.13, 39.02)	0.17
A_23_P54088	*OR4K17*	olfactory receptor family 4 subfamily K member 17	16.96 (−2.58, 49.08)	0.17
A_23_P325661	*ZNF134*	zinc finger protein 134	16.46 (−2.12, 41.68)	0.17
A_23_P381945	*KRT7*	keratin 7	15.68 (−0.88, 39.02)	0.17
A_23_P427136	*TSSK1B*	testis specific serine kinase 1B	15.64 (−4.65, 50.75)	0.22
A_23_P154522	*MTA3*	metastasis associated 1 family member 3	15.31 (−8.64, 57.31)	0.29
A_24_P344295	*RNF167*	ring finger protein 167	15.04 (−2.19, 39.49)	0.17
A_23_P9209	*NIPSNAP3B*	nipsnap homolog 3B	14.56 (−12.47, 55.74)	0.29
A_23_P135787	*GOLGB1*	golgin B1	14.37 (−8.34, 51.15)	0.27
A_24_P416301	*FOXK2*	forkhead box K2	13.92 (−23.25, 71.77)	0.47
A_24_P145629	*SERINC2*	serine incorporator 2	13.90 (−7.15, 46.81)	0.23
A_23_P306755	*CRYAA*	crystallin alpha A	13.80 (−1.50, 39.97)	0.17
A_24_P169688	*MICB*	MHC class I polypeptide-related sequence B	13.50 (1.03, 29.72)	0.17
A_23_P39454	*ZNF556*	zinc finger protein 556	13.44 (−3.22, 42.61)	0.21
A_32_P134968	*SPTB*	spectrin beta, erythrocytic	13.43 (−0.03, 36.34)	0.17
A_32_P165116	*DNAAF10*	dynein axonemal assembly factor 10	13.04 (−1.58, 34.74)	0.17
A_24_P323425	*DZANK1*	double zinc ribbon and ankyrin repeat domains 1	12.97 (−10.07, 49.64)	0.29
A_24_P173754	*C1orf21*	chromosome 1 open reading frame 21	12.58 (−2.55, 34.36)	0.17
A_23_P332413	*SLFN13*	schlafen family member 13	12.36 (−2.49, 32.56)	0.17
A_23_P170352	*MRPL12*	mitochondrial ribosomal protein L12	12.05 (−0.41, 31.74)	0.17
A_24_P77941	*VPS50*	VPS50 subunit of EARP/GARPII complex	−11.16 (−32.04, 1.69)	0.17
A_24_P384119	*IGHV3OR16-13*	immunoglobulin heavy variable 3/OR16-13 (non-functional)	−11.35 (−31.93, 0.65)	0.17
A_23_P500010	*KLK12*	kallikrein related peptidase 12	−12.04 (−34.86, 1.61)	0.17
A_23_P210400	*KCNQ2*	potassium voltage-gated channel subfamily Q member 2	−12.25 (−34.90, 1.95)	0.17
A_24_P114255	*MBOAT2*	membrane bound O-acyltransferase domain containing 2	−12.54 (−34.35, 0.66)	0.17
A_24_P77219	*ARID1A*	AT-rich interaction domain 1A	−12.58 (−36.30, 1.68)	0.17
A_24_P161604	*RPL21P120*	ribosomal protein L21 pseudogene 120	−13.40 (−36.42, −0.43)	0.17
A_24_P919084	*SLC22A16*	solute carrier family 22 member 16	−14.34 (−36.44, −1.04)	0.17
A_23_P94840	*DYNLRB2*	dynein light chain roadblock-type 2	−17.57 (−41.44, −1.73)	0.17
A_24_P299663	*ZBTB18*	zinc finger and BTB domain containing 18	−21.07 (−44.19, −4.27)	0.17

Genes highlighted in red represent genes that are components of metabolism-related pathways. Abbreviations: ACMEs, average causal mediation effects; MDCs, metabolism-disrupting chemicals; *p,p’*-DDE, dichlorodiphenyldichloroethylene; PCB-153, polychlorinated biphenyl 153; PFOA, perfluorooctanoic acid; PFOS, perfluorooctane sulfonic acid; FDR, false discovery rate.

**Table 4:** The ACMEs of an MDC on birth weight via overlapping pathways.

		*p,p’*-DDE			
Pathway	Category	Gene Size	Variance by PC1 (%)	ACME (95% CI, g)	FDR
Olfactory transduction	OS (Sensory system)	143	40	−2.21 (−12.41, 3.39)	0.64
Taste transduction	OS (Sensory system)	59	39	−1.49 (−10.67, 3.65)	0.64
Ribosome	GIP (Translation)	126	49	−5.16 (−18.73, 4.64)	0.64
RNA transport	GIP (Translation)	149	38	−1.75 (−11.13, 3.53)	0.64
		**PCB-153**			
**Pathway**	**Category**	**Gene Size**	**Variance by PC1 (%)**	**ACME (95% CI, g)**	**FDR**
Ribosome	GIP (Translation)	126	49	−1.10 (−12.15, 8.90)	0.85
Fanconi anemia pathway	GIP (Replication and repair)	46	45	2.80 (−9.06, 17.83)	0.85
Mismatch repair	GIP (Replication and repair)	22	56	3.42 (−8.67, 18.67)	0.85
		**PFOA**			
**Pathway**	**Category**	**Gene Size**	**Variance by PC1 (%)**	**ACME (95% CI, g)**	**FDR**
Olfactory transduction	OS (Sensory system)	143	40	3.11 (−9.18, 19.74)	0.78
NLRI	EIP (Signaling molecules and interaction)	219	34	2.99 (−8.60, 18.53)	0.78
Spliceosome	GIP (Transcription)	124	53	2.89 (−9.81, 19.16)	0.78
Proteasome	GIP (Folding, sorting and degradation)	43	57	2.57 (−10.08, 18.81)	0.78
Autophagy	CP (Transport and catabolism)	31	37	1.76 (−9.39, 17.44)	0.78
PPIER	GIP (Folding, sorting and degradation)	156	41	2.92 (−8.66, 19.27)	0.78
		**PFOS**			
**Pathway**	**Category**	**Gene Size**	**Variance by PC1 (%)**	**ACME (95% CI, g)**	**FDR**
Spliceosome	GIP (Transcription)	124	53	2.44 (−5.78, 13.78)	0.71
Fanconi anemia pathway	GIP (Replication and repair)	46	45	1.97 (−7.21, 13.64)	0.71
Mismatch repair	GIP (Replication and repair)	22	56	2.63 (−6.69, 14.38)	0.71

ACMEs were estimated by summarizing feature intensities with principal component, corresponding to about 50% of transcription variance in the gene set from each pathway. Abbreviations: ACMEs, average causal mediation effects; MDCs, metabolism-disrupting chemicals; *p,p’*-DDE, dichlorodiphenyldichloroethylene; PCB-153, polychlorinated biphenyl 153; PFOA, perfluorooctanoic acid; PFOS, perfluorooctane sulfonic acid; FDR, false discovery rate; NLRI, Neuroactive ligand–receptor interaction; PPIER, Protein processing in endoplasmic reticulum; OS, Organismal Systems; GIP, Genetic Information Processing; EIP, Environmental Information Processing; CP, Cellular Processess.

## Data Availability

Data available on request due to privacy restrictions.

## Supplementary Materials

The supporting information can be downloaded at: https://www.mdpi.com/article/10.3390/ijms24087607/s1.
