# Supplementary material for: Prenatal Exposure to Metabolism-Disrupting Chemicals, Cord Blood Transcriptome Perturbations, and Birth Weight in a Belgian Birth Cohort
Source: Int J Mol Sci. 2023 Apr 20;24(8):7607. doi: 10.3390/ijms24087607 (PMC10141364; doi:10.3390/ijms24087607)
Supplement: Supplementary file 1 [file ijms-24-07607-s001.zip › ijms-2279366-supplementary.pdf]

# Supplementary Materials

## Prenatal exposure to metabolism-disrupting chemicals, cord blood transcriptome perturbations, and birth weight in a Belgian birth cohort

Anran Cai, Lützen Portengen, Gökhan Ertaylan, Juliette Legler, Roel Vermeulen, Virissa Lenters, Sylvie Remy

**Table of contents**

**Table S1.** The concentrations of MDCs measured in cord blood among 193 newborns.

**Table S2.** Number of features associated with MDCs at different significance levels (sensitivity analysis: gestational age-unadjusted).

**Table S3.** The individual associations of gene expression with MDCs or birth weight.

**Table S4.** Enriched KEGG pathways associated with MDCs and birth weight at FDR < 0.05.

**Figure S1.** Volcano plots of features in response to MDCs and birth weight.

**Figure S2.** DAG of authors' conception of the associations between prenatal MDC exposures, covariates, and (a) cord blood transcriptome and (b) birth weight among infants born at term.

**Table S1.** The concentrations of MDCs measured in cord blood among 193 newborns.

| | Missing, *n* (%) | LOQ (ng/L) | > LOQ (%) | Median (P25-P75) (ng/L) | Median [a] (P25-P75) (ng/g lipid) |
|---|---|---|---|---|---|
| *p,p′*-DDE | 2 (1) | 20 | 100 | 157 (91-255) | 75.0 (45.8-134.0) |
| PCB-153 | 2 (1) | 20 | 97 | 54 (40-85) | 28.7 (18.7-39.4) |
| PFOA | 25 (13) | 300 | 100 | 1600 (1100-2100) | |
| PFOS | 25 (13) | 300 | 100 | 2700 (1700-3700) | |

[a] Lipophilic chemicals *p,p′*-DDE and PCB-153 were lipid-standardized and expressed in ng/g lipid.

Abbreviations: MDCs, metabolism-disrupting chemicals; *p,p′*-DDE, dichlorodiphenyldichloroethylene; PCB-153, polychlorinated biphenyl 153; PFOA, perfluorooctanoic acid; PFOS, perfluorooctane sulfonic acid; LOQ, limit of quantification; P, percentile.

**Table S2.** Number of features associated with MDCs at different significance levels (sensitivity analysis: gestational age-unadjusted).

| | **FDR < 0.05** | **FDR < 0.20** | ***p*-value < 0.01** | ***p*-value < 0.05** |
|---|---|---|---|---|
| *p,p,′*-DDE | 0 | 0 | 146 | 837 |
| PCB-153 | 0 | 0 | 78 | 634 |
| PFOA | 0 | 0 | 33 | 375 |
| PFOS | 0 | 0 | 79 | 631 |

Abbreviations: MDCs, metabolism-disrupting chemicals; *p,p′*-DDE, dichlorodiphenyldichloroethylene; PCB-153, polychlorinated biphenyl 153; PFOA, perfluorooctanoic acid; PFOS, perfluorooctane sulfonic acid; FDR, false discovery rate.

**Table S3.** The individual associations of gene expression with MDCs or birth weight, β (*p*-value).

| ProbeID | GeneSymbol | *p,p'*-DDE [a] | PCB-153 [a] | PFOA [a] | PFOS [a] | Birth weight [b] |
|---|---|---|---|---|---|---|
| A_23_P90163 | BCAT2 | -0.07 (0.04) | | | | 128.82 (0.04) |
| A_23_P129322 | IVD | | -0.17 (0.04) | | | -98.48 (0.01) |
| A_32_P74075 | SLC25A16 | | -0.10 (0.02) | | | 208.48 (0.01) |
| A_23_P357229 | HAS3 | | | 0.04 (0.23) | | 79.70 (0.04) |
| A_24_P114255 | MBOAT2 | | | | 0.08 (0.03) | -153.87 (0.04) |

[a] The effect estimate β refers to $log_2$-fold change in gene expression per unit increase in log(MDC).

[b] The effect estimate β refers to birth weight change (g) per $log_2$-fold increase in gene expression.

Abbreviations: MDCs, metabolism-disrupting chemicals; *p,p'*-DDE, dichlorodiphenyldichloroethylene; PCB-153, polychlorinated biphenyl 153; PFOA, perfluorooctanoic acid; PFOS, perfluorooctane sulfonic acid; FDR, false discovery rate.

**Table S4.** Enriched KEGG pathways associated with MDCs and birth weight at FDR < 0.05.

| Rank | Pathway | Category | NES | FDR | Effective/total gene size |
|---|---|---|---|---|---|
| | | ***p,p′*-DDE** | | | |
| **Rank** | **Pathway** | **Category** | **NES** | **FDR** | **Effective/total gene size** |
| 1 | Ribosome | GIP (Translation) | 2.18 | 0.00 | 76/126 |
| 2 | Allograft rejection | HD (Immune disease) | 2.08 | 0.01 | 21/34 |
| 3 | RNA degradation | GIP (Folding, sorting and degradation) | 2.05 | 0.01 | 44/74 |
| 4 | Intestinal immune network for IgA production | OS (Immune system) | 1.93 | 0.02 | 15/38 |
| 5 | T cell receptor signaling pathway | OS (Immune system) | 1.93 | 0.02 | 71/91 |
| 6 | RNA transport | GIP (Translation) | 1.92 | 0.02 | 74/149 |
| 7 | Th1 and Th2 cell differentiation | OS (Immune system) | 1.92 | 0.02 | 34/81 |
| 8 | Th17 cell differentiation | OS (Immune system) | 1.89 | 0.02 | 38/101 |
| 9 | Hematopoietic cell lineage | OS (Immune system) | 1.86 | 0.02 | 28/87 |
| 10 | Glycosaminoglycan biosynthesis | Metabolism (Glycan biosynthesis and metabolism) | 1.81 | 0.04 | 7/21 |
| 11 | Inflammatory bowel disease (IBD) | HD (Immune disease) | 1.80 | 0.04 | 22/59 |
| 12 | Platelet activation | OS (Immune system) | 1.80 | 0.03 | 32/109 |
| 13 | Autoimmune thyroid disease | HD (Immune disease) | 1.79 | 0.03 | 20/40 |
| 14 | Antigen processing and presentation | OS (Immune system) | 1.79 | 0.03 | 41/71 |
| 15 | Ribosome biogenesis in eukaryotes | GIP (Translation) | 1.75 | 0.04 | 35/72 |
| 1 | Olfactory transduction | OS (Sensory system) | -2.17 | 0.00 | 61/143 |
| 2 | Taste transduction | OS (Sensory system) | -1.95 | 0.03 | 33/59 |
| | | **PCB-153** | | | |
| **Rank** | **Pathway** | **Category** | **NES** | **FDR** | **Effective/total gene size** |
| 1 | Ribosome | GIP (Translation) | -2.33 | < 2.2e-16 | 85/126 |
| 2 | Mismatch repair | GIP (Replication and repair) | -2.01 | 0.01 | 12/22 |
| 3 | Fanconi anemia pathway | GIP (Replication and repair) | -1.84 | 0.04 | 23/46 |
| | | **PFOA** | | | |
| **Rank** | **Pathway** | **Category** | **NES** | **FDR** | **Effective/total gene size** |
| 1 | Metabolism of xenobiotics by cytochrome P450 | Metabolism (Xenobiotics biodegradation and metabolism) | 2.22 | < 2.2e-16 | 25/51 |
| 2 | Drug metabolism | Metabolism (Xenobiotics biodegradation and metabolism) | 2.16 | < 2.2e-16 | 23/47 |
| 3 | Olfactory transduction | OS (Sensory system) | 1.88 | 0.02 | 64/143 |
| 4 | Neuroactive ligand-receptor interaction | EIP (Signaling molecules and interaction) | 1.83 | 0.03 | 79/219 |

| | | | | | |
|---|---|---|---|---|---|
| 1 | Antigen processing and presentation | OS (Immune system) | -2.57 | < 2.2e-16 | 38/71 |
| 2 | Natural killer cell mediated cytotoxicity | OS (Immune system) | -2.53 | < 2.2e-16 | 32/107 |
| 3 | Graft-versus-host disease | HD (Immune disease) | -2.43 | < 2.2e-16 | 17/36 |
| 4 | Th1 and Th2 cell differentiation | OS (Immune system) | -2.3 | < 2.2e-16 | 27/81 |
| 5 | Spliceosome | GIP (Transcription) | -2.06 | 0.00 | 66/124 |
| 6 | Viral myocarditis | HD (Cardiovascular disease) | -2.04 | 0.00 | 19/53 |
| 7 | Type I diabetes mellitus | HD (Endocrine and metabolic disease) | -2.02 | 0.00 | 16/38 |
| 8 | Human immunodeficiency virus 1 infection | HD (Infectious disease: viral) | -1.97 | 0.00 | 47/188 |
| 9 | Colorectal cancer | HD (Cancer: specific types) | -1.95 | 0.01 | 35/80 |
| 10 | Cellular senescence | CP (Cell growth and death) | -1.94 | 0.01 | 57/148 |
| 11 | Apoptosis | CP (Cell growth and death) | -1.92 | 0.01 | 44/125 |
| 12 | Th17 cell differentiation | OS (Immune system) | -1.89 | 0.01 | 28/101 |
| 13 | Measles | HD (Infectious disease: viral) | -1.86 | 0.01 | 39/111 |
| 14 | Allograft rejection | HD (Immune disease) | -1.84 | 0.02 | 14/34 |
| 15 | Herpes simplex infection | HD (Infectious disease: viral) | -1.81 | 0.02 | 59/164 |
| 16 | Salmonella infection | HD (Infectious disease: viral) | -1.80 | 0.02 | 36/74 |
| 17 | B cell receptor signaling pathway | OS (Immune system) | -1.76 | 0.03 | 28/69 |
| 18 | Autophagy | CP (Transport and catabolism) | -1.75 | 0.03 | 48/121 |
| 19 | FoxO signaling pathway | EIP (Signal transduction) | -1.73 | 0.04 | 44/115 |
| 20 | Epstein-Barr virus infection | HD (Infectious disease: viral) | -1.72 | 0.04 | 86/183 |
| 21 | Protein processing in endoplasmic reticulum | GIP (Folding, sorting and degradation) | -1.70 | 0.04 | 54/156 |
| 22 | Proteasome | GIP (Folding, sorting and degradation) | -1.69 | 0.04 | 28/43 |
| 23 | Pathogenic Escherichia coli infection | HD (Infectious disease: bacterial) | -1.69 | 0.04 | 20/47 |

**PFOS**

| Rank | Pathway | Category | NES | FDR | Effective/total gene size |
|---|---|---|---|---|---|
| 1 | Leishmaniasis | HD (Infectious disease: parasitic) | -2.16 | 0.01 | 41/68 |
| 2 | Natural killer cell mediated cytotoxicity | OS (Immune system) | -2.04 | 0.03 | 49/107 |
| 3 | Antigen processing and presentation | OS (Immune system) | -2.02 | 0.02 | 34/71 |
| 4 | Spliceosome | GIP (Transcription) | -2.02 | 0.02 | 70/124 |
| 5 | B cell receptor signaling pathway | OS (Immune system) | -1.97 | 0.02 | 33/69 |
| 6 | NOD-like receptor signaling pathway | OS (Immune system) | -1.97 | 0.02 | 59/145 |

| Rank | Pathway | Category | NES | FDR | Effective/total gene size |
|---|---|---|---|---|---|
| 7 | Salmonella infection | HD (Infectious disease: viral) | -1.96 | 0.02 | 36/74 |
| 8 | Graft-versus-host disease | HD (Immune disease) | -1.92 | 0.02 | 12/36 |
| 9 | Phagosome | CP (Transport and catabolism) | -1.89 | 0.02 | 34/137 |
| 10 | Amino sugar and nucleotide sugar metabolism | Metabolism (Carbohydrate metabolism) | -1.88 | 0.02 | 14/42 |
| 11 | Osteoclast differentiation | OS (Development and regeneration) | -1.83 | 0.03 | 46/115 |
| 12 | Acute myeloid leukemia | HD (Cancer: specific types) | -1.82 | 0.03 | 23/61 |
| 13 | Influenza A | HD (Infectious disease: viral) | -1.81 | 0.03 | 52/147 |
| 14 | Fanconi anemia pathway | GIP (Replication and repair) | -1.79 | 0.04 | 16/46 |
| 15 | Viral myocarditis | HD (Cardiovascular disease) | -1.78 | 0.04 | 14/53 |
| 16 | Mismatch repair | GIP (Replication and repair) | -1.77 | 0.04 | 11/22 |
| 17 | Pancreatic cancer | HD (Cancer: specific types) | -1.77 | 0.04 | 26/69 |
| 18 | Type I diabetes mellitus | HD (Endocrine and metabolic disease) | -1.75 | 0.04 | 22/38 |
| 19 | Kaposi sarcoma-associated herpesvirus infection | HD (Infectious disease: viral) | -1.72 | 0.04 | 58/165 |
| 20 | Pathogenic Escherichia coli infection | HD (Infectious disease: bacterial) | -1.70 | 0.04 | 22/47 |

| | | **Birth weight** | | | |
|---|---|---|---|---|---|
| **Rank** | **Pathway** | **Category** | **NES** | **FDR** | **Effective/total gene size** |
| 1 | Olfactory transduction | OS (Sensory system) | 2.75 | < 2.2e-16 | 82/143 |
| 2 | Neuroactive ligand-receptor interaction | EIP (Signaling molecules and interaction) | 2.24 | 0.00 | 115/219 |
| 3 | Nicotine addiction | HD (Substance dependence) | 2.10 | 0.00 | 17/32 |
| 4 | Taste transduction | OS (Sensory system) | 2.04 | 0.00 | 25/59 |
| 5 | Cytokine-cytokine receptor interaction | EIP (Signaling molecules and interaction) | 1.95 | 0.01 | 95/221 |
| 6 | Dilated cardiomyopathy (DCM) | HD (Cardiovascular disease) | 1.85 | 0.02 | 23/83 |
| 1 | Ribosome | GIP (Translation) | -2.78 | < 2.2e-16 | 91/126 |
| 2 | Spliceosome | GIP (Transcription) | -2.62 | < 2.2e-16 | 75/124 |
| 3 | Oxidative phosphorylation | Metabolism (Energy metabolism) | -2.43 | < 2.2e-16 | 52/110 |
| 4 | Proteasome | GIP (Folding, sorting and degradation) | -2.39 | < 2.2e-16 | 31/43 |
| 5 | Cell cycle | CP (Cell growth and death) | -2.30 | < 2.2e-16 | 70/117 |
| 6 | Fanconi anemia pathway | GIP (Replication and repair) | -2.28 | < 2.2e-16 | 22/46 |
| 7 | DNA replication | GIP (Replication and repair) | -2.14 | 0.00 | 18/35 |
| 8 | Non-alcoholic fatty liver disease (NAFLD) | HD (Endocrine and metabolic disease) | -2.14 | 0.00 | 54/131 |
| 9 | Nucleotide excision repair | GIP (Replication and repair) | -2.13 | 0.00 | 22/42 |

| | | | | | |
|---|---|---|---|---|---|
| 10 | Huntington disease | HD (Neurodegenerative disease) | -2.09 | 0.00 | 80/166 |
| 11 | Mismatch repair | GIP (Replication and repair) | -2.05 | 0.00 | 15/22 |
| 12 | Parkinson disease | HD (Neurodegenerative disease) | -2.01 | 0.00 | 49/116 |
| 13 | RNA transport | GIP (Translation) | -2.00 | 0.00 | 65/149 |
| 14 | Autophagy | CP (Transport and catabolism) | -1.91 | 0.01 | 13/31 |
| 15 | Homologous recombination | GIP (Replication and repair) | -1.91 | 0.01 | 23/36 |
| 16 | Autophagy_1 | CP (Transport and catabolism) | -1.85 | 0.01 | 32/121 |
| 17 | Thermogenesis | OS (Environmental adaptation) | -1.85 | 0.01 | 88/196 |
| 18 | Alzheimer disease | HD (Neurodegenerative disease) | -1.85 | 0.01 | 50/152 |
| 19 | Protein processing in endoplasmic reticulum | GIP (Folding, sorting and degradation) | -1.83 | 0.01 | 73/156 |
| 20 | RNA polymerase | GIP (Transcription) | -1.78 | 0.02 | 14/27 |
| 21 | mRNA surveillance pathway | GIP (Translation) | -1.77 | 0.02 | 34/84 |
| 22 | Cysteine and methionine metabolism | Metabolism (Amino acid metabolism) | -1.77 | 0.02 | 13/40 |
| 23 | Protein export | GIP (Folding, sorting and degradation) | -1.72 | 0.03 | 15/23 |
| 24 | Sulfur metabolism | Metabolism (Energy metabolism) | -1.70 | 0.03 | 8/12 |
| 25 | Valine, leucine and isoleucine degradation | Metabolism (Energy metabolism) | -1.69 | 0.04 | 16/43 |
| 26 | Ubiquitin mediated proteolysis | GIP (Folding, sorting and degradation) | -1.68 | 0.04 | 50/126 |
| 27 | Fatty acid biosynthesis | Metabolism (Lipid metabolism) | -1.67 | 0.04 | 16/35 |

The highlighted cells in ranks columns refer to positive (blue) and inverse (orange) associations and the red font in pathway columns denote metabolism-related pathways. Abbreviations: MDCs, metabolism-disrupting chemicals; *p,p′*-DDE, dichlorodiphenyldichloroethylene; PCB-153, polychlorinated biphenyl 153; PFOA, perfluorooctanoic acid; PFOS, perfluorooctane sulfonic acid; KEGG, Kyoto Encyclopedia of Genes and Genomes; NES, normalized enrichment score; FDR, false discovery rate; GIP, Genetic Information Processing; OS, Organismal Systems; EIP, Environmental Information Processing; CP, Cellular Processes; HD, Human Diseases.

(a) *p,p′*-DDE

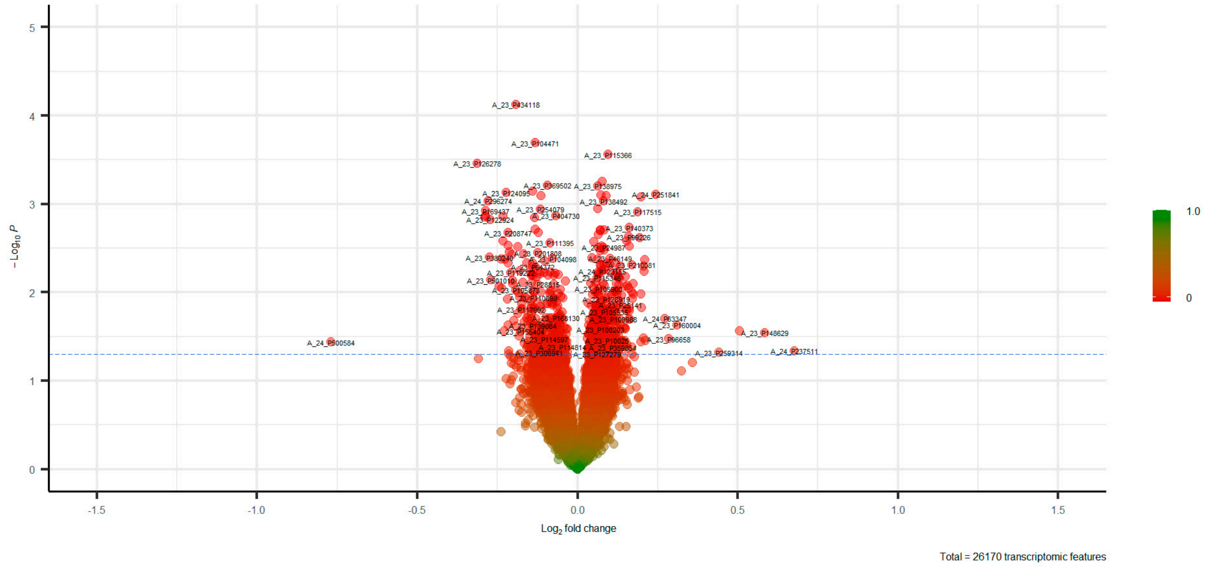


Sensitivity analysis (gestational age unadjusted)

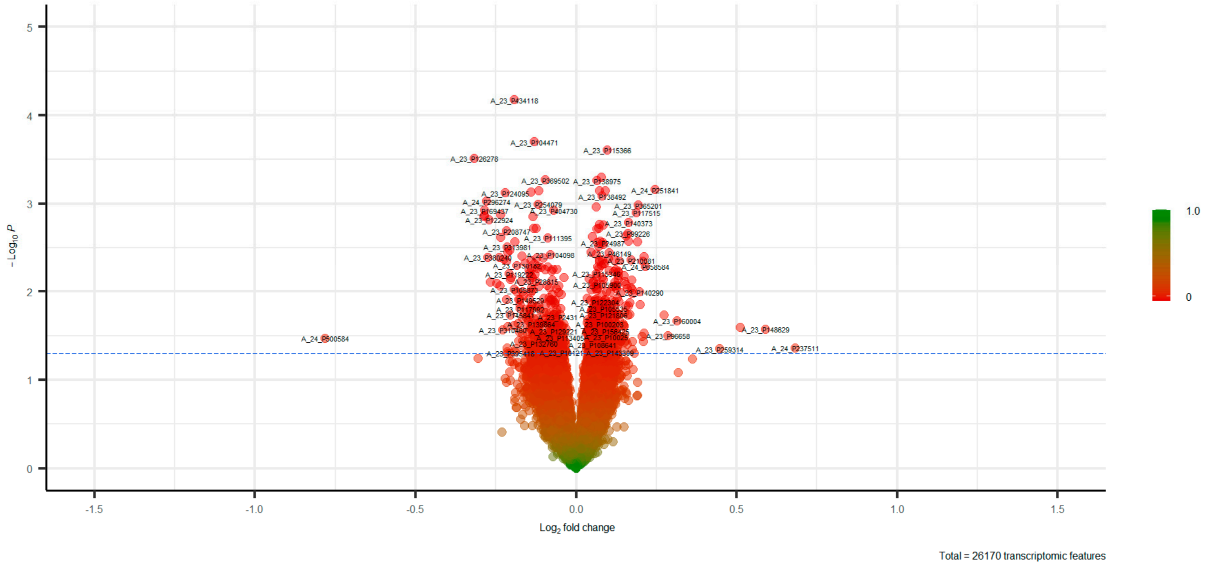


**Figure S1.** Volcano plots of features in response to MDCs and birth weight.
The points above the blue line show differentially expressed features with *p*-value < 0.05; the points below the blue line show no differential gene expression with *p*-value < 0.05.
Abbreviations: MDCs, metabolism-disrupting chemicals; *p,p′*-DDE, dichlorodiphenyldichloroethylene; PCB-153, polychlorinated biphenyl 153; PFOA, perfluorooctanoic acid; PFOS, perfluorooctane sulfonic acid.

(b) PCB-153

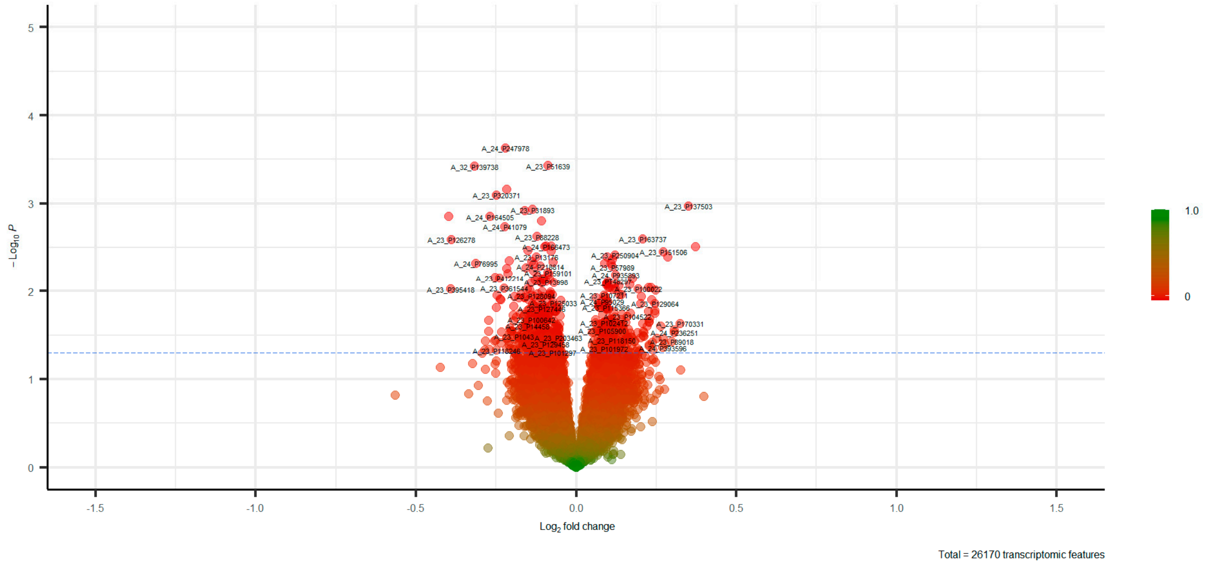


Sensitivity analysis (gestational age unadjusted)

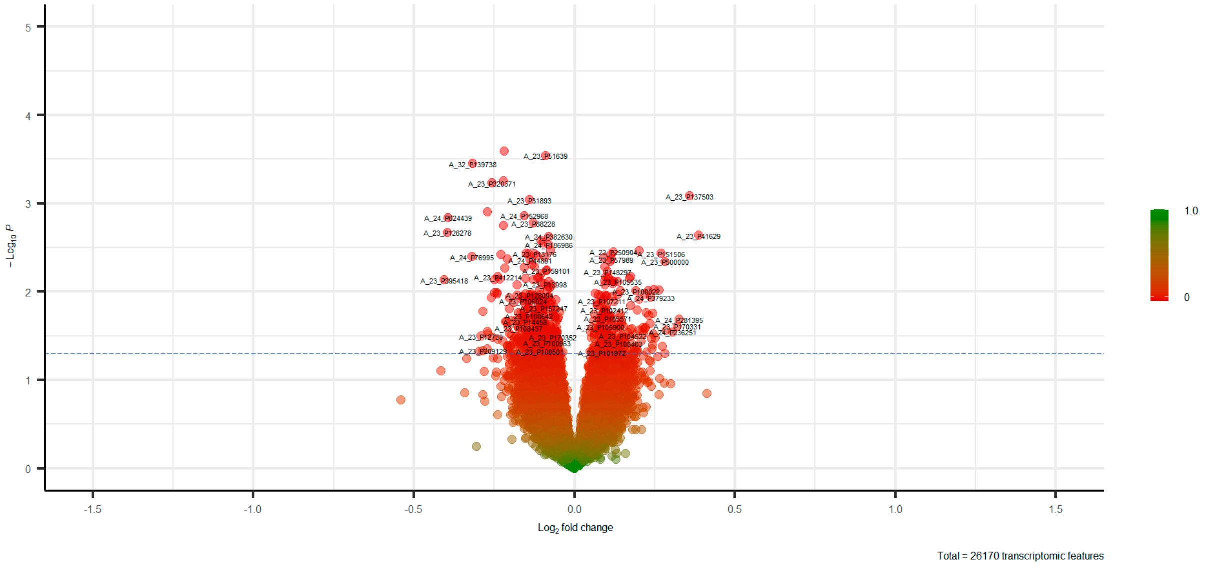


**Figure S1** continued

(c) PFOA

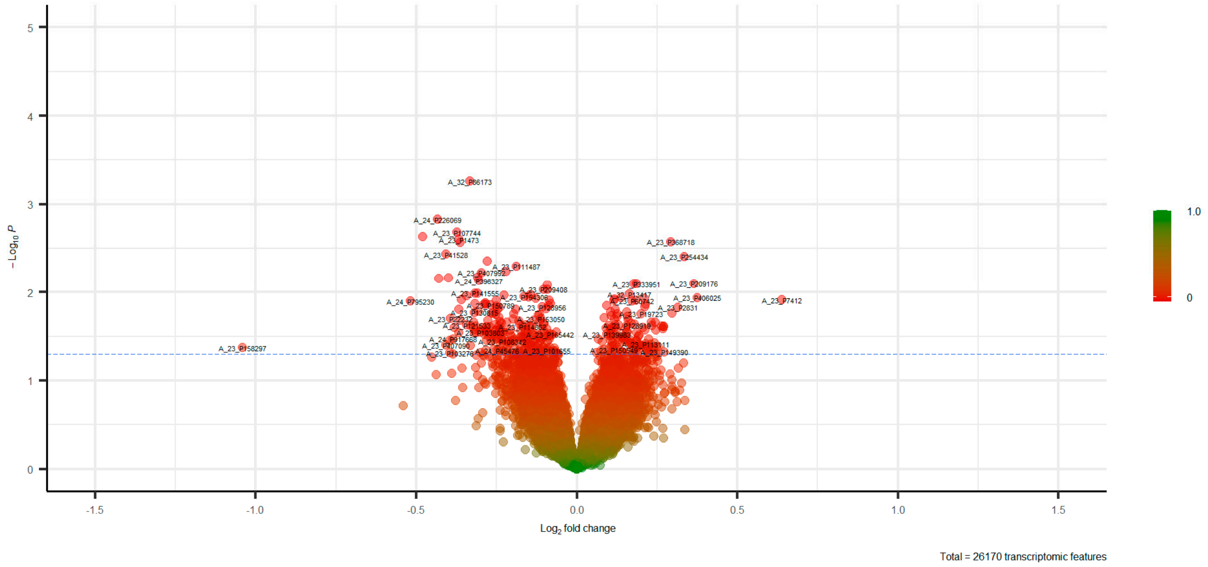


Sensitivity analysis (gestational age unadjusted)

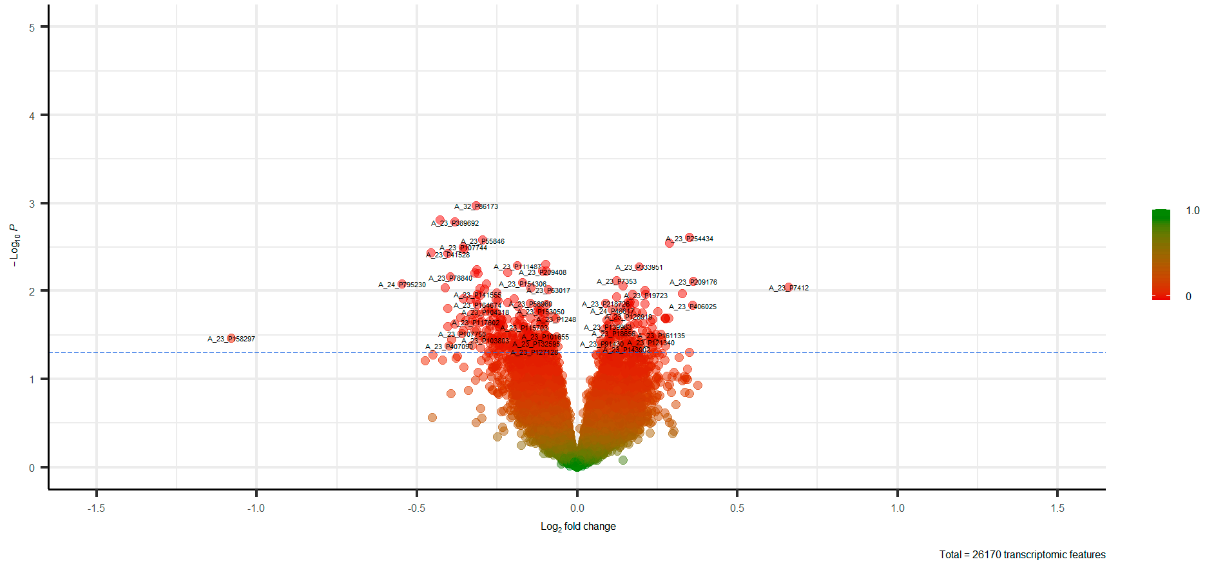


**Figure S1** continued

(d) PFOS

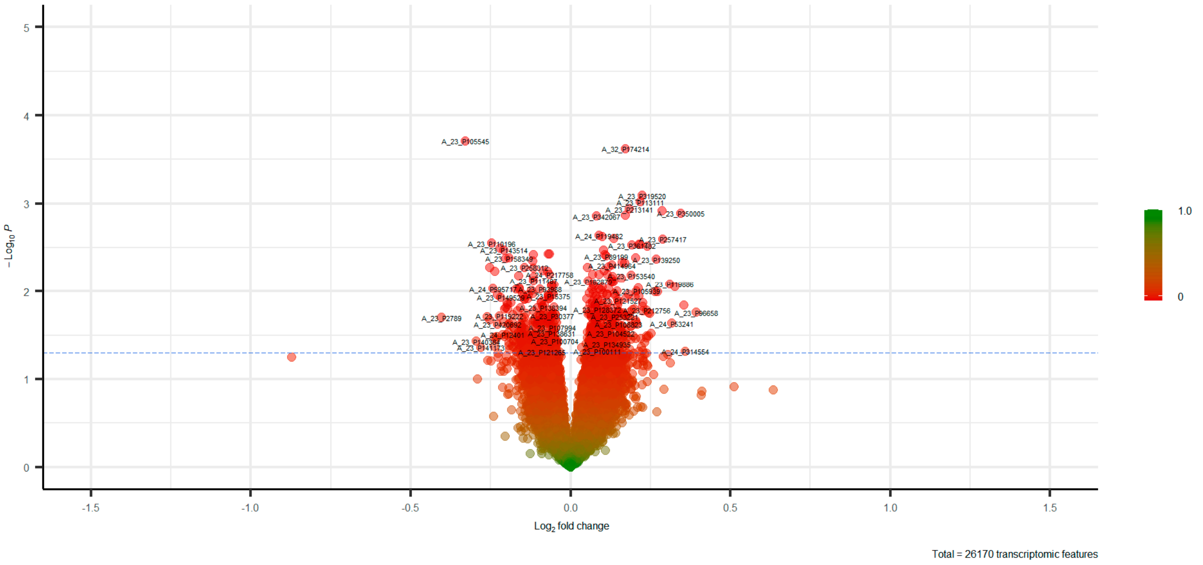


Sensitivity analysis (gestational age unadjusted)

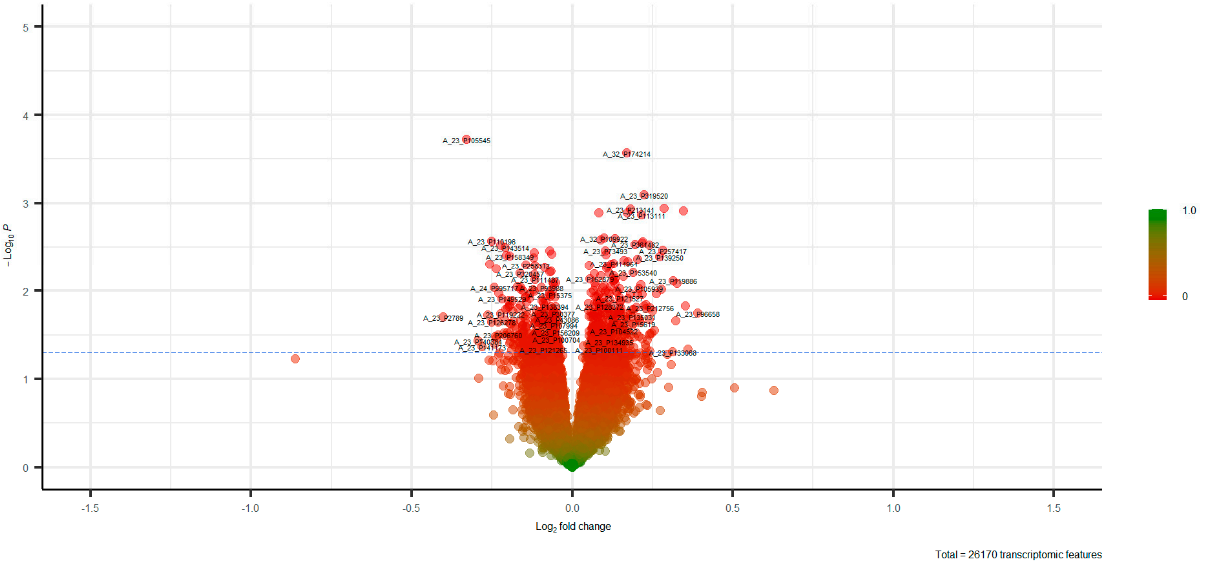


**Figure S1** continued

(e) Birth weight

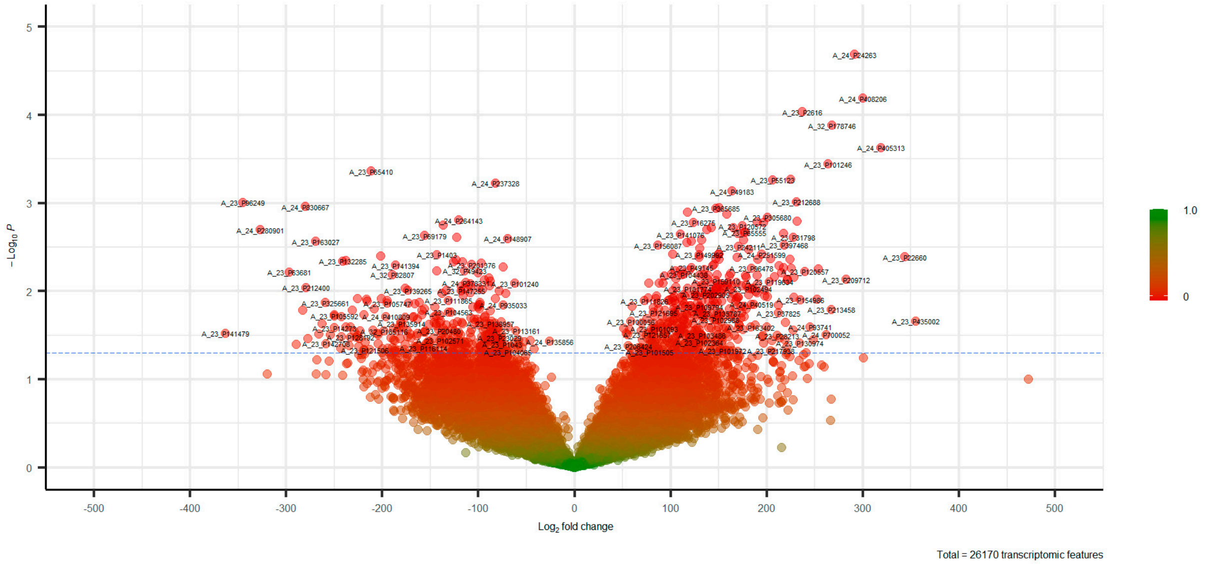


**Figure S1** continued

a)

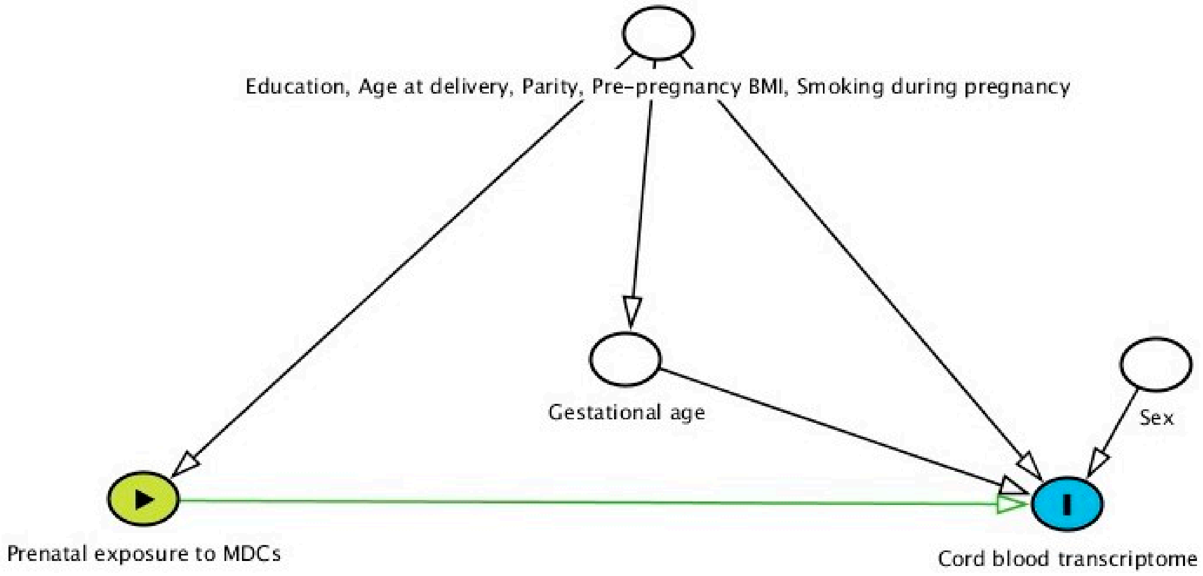


b)

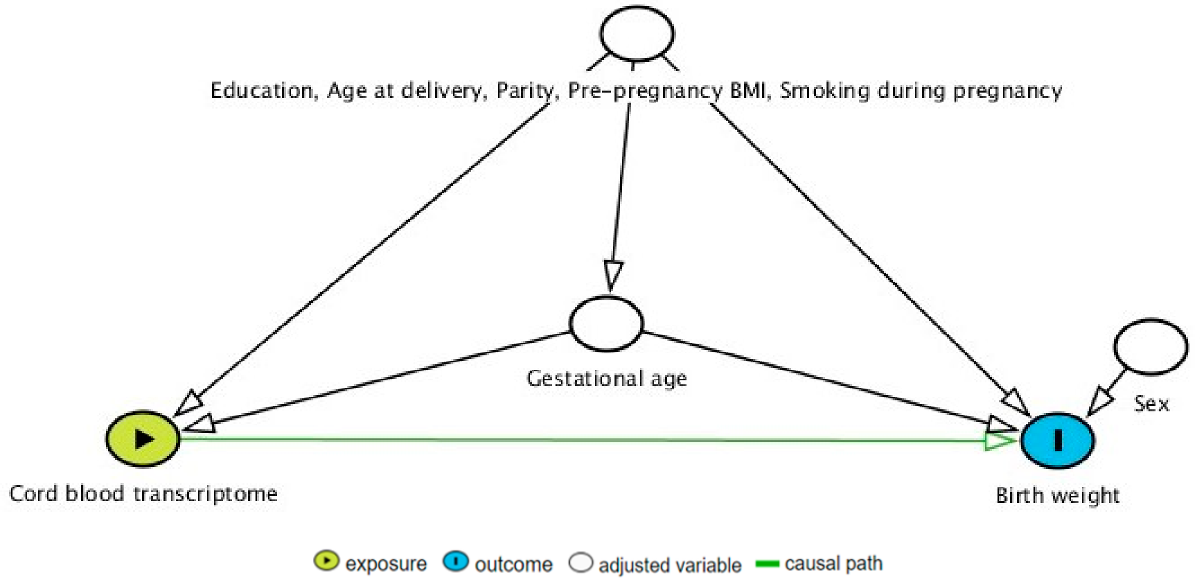


**Figure S2.** DAG of authors' conception of the associations between prenatal MDC exposures, covariates, and (a) cord blood transcriptome and (b) birth weight among infants born at term.

Abbreviations: DAG, Directed acyclic graphs; MDC, metabolism-disrupting chemical; *p,p'*-DDE, dichlorodiphenyldichloroethylene; PCB-153, polychlorinated biphenyl 153; PFOA, perfluorooctanoic acid; PFOS, perfluorooctane sulfonic acid.
